# Spatial Multi-Source Information Fusion Localization Algorithm in Non-Line-of-Sight Environments

**DOI:** 10.3390/s23073482

**Published:** 2023-03-27

**Authors:** Jianhui Wang, Jingjing Li, Weijia Cui, Qing Liu

**Affiliations:** National Digital Switching System Engineering & Technological Research Center, Zhengzhou 450001, China

**Keywords:** wireless localization, non-line-of-sight (NLOS), information fusion, propagation path, localization accuracy

## Abstract

In this paper, a high-accuracy localization problem under a complex non-line-of-sight (NLOS) condition is addressed by a new method that utilizes multiple NLOS paths to improve localization accuracy, as opposed to the traditional method of suppressing them. The spatial multi-path array fusion localization model is presented and analyzed, followed by an angle-of-arrival (AOA) and time-of-arrival (TOA) algorithm based on spatial multi-information fusion that seeks to improve localization accuracy. Multi-path of spatial signals, measurement of the multi-element antenna, and geographic environment information are integrated into the proposed method for localization optimization. Simulation experiments were carried out, and the results revealed that the proposed algorithm is capable of making full use of spatial multi-location information for localization, thus improving the accuracy of localization in a the NLOS environment effectively and increasing the locatable probability of complex environment localization applications.

## 1. Introduction

In the wireless localization system, when non-line-of-sight (NLOS) propagation occurs between the target and localization station, deviation of the angle-of-arrival (AOA) or time-of-arrival (TOA) can occur due to the effect of terrain, barrier, and geography, which is called a NLOS error. NLOS and multi-path interference are the major error sources in positioning [1]. NLOS errors are becoming an increasingly serious issue due to the rapid expansion of cities and the increasing number of buildings and flat surface reflectors [1,2]. NLOS errors have far-reaching implications. They not only affects the value of the time delay, but they also have a severe impact on the values of the angle and power, which has become the main issue that restricts localization accuracy. In addition, the signal arriving at the receiver antenna can arrive through multi-paths (reflection, refraction, scattering, etc.), and the superposition of those signals can produce multi-path fading. This can lead to a decrease in signal strength, as well as a distortion of the signal waveform. The traditional time delay estimation algorithm struggles to distinguish the multi-paths, and leads to measurement errors when the difference in arrival times of the multi-paths is slight. Multi-paths and NLOS errors lead to serious interference, especially in the urban environment, where multi-paths can create Rayleigh fading, severely affects the quality of communications, and, even worse, the accuracy of timing and ranging measurements [3]. Multi-path effects and signal blockages in an urban canyon can also lead to the inaccuracy and unavailability of global navigation satellite systems [4]. Doppler frequency offset estimation is also disturbed by multi-path interference, leading to carrier phase perturbations [5]. As multi-path interference is present in most localization scenarios, it severely affects the accuracy of positioning.

Currently, there are two approaches for reducing the NLOS error in localization algorithms. The first approach considers the NLOS error as the solution of the localization algorithm when either the target is moving slowly with slow environmental changes or when the target is moving quickly with short measurement time. The second approach models the observed signals and NLOS error as a specific probability distribution, such as exponential, uniform, Gaussian, or delta distribution, depending on the channel condition. Research on NLOS errors is divided into two categories: NLOS identification, and mitigation without identification. The first category aims to detect and identify the presence of an NLOS error in the received signal. Once the NLOS error is identified, it can be mitigated by using an algorithm. For example, Wylie proposed a detecting algorithm based on the standard deviation of noise [6]. In another study [7], a Markov chain model of LOS/NLOS based on the assumption of NLOS intermittency was developed to identify and mitigate NLOS errors. An NLOS identification and mitigation algorithm based on the distributional characteristics of NLOS was proposed in [8], with the correctness of the results having a directly impact on the performance of NLOS error mitigation algorithms. The second category focuses on mitigating the effects of NLOS errors using the corresponding algorithms by taking advantage of their characteristics rather than identifying their presence. For example, the authors in [9] proposed a semidefinite programming-based algorithm to mitigate the NLOS error without the requirement of NLOS information or identification of the NLOS error. 

For ground-based NLOS scenarios, the location of reflectors (e.g., tall buildings, mountains, trees) is invariable. Thus, geographical information data or building data can be introduced to algorithms as reflectors to assist in positioning, increasing the available target location information, which can improve localization performance. In order to improve the localization accuracy, we need to fully account for the errors existing in the localization model. In practical applications, the position of the reflector may have errors, especially the uncertainly of the target position and status, which makes the position of the reflection points on the same reflection change over time. To address this issue, it is necessary to further integrate array metadata, multi-path data, and other information for joint optimization. Information fusion from multi-sources can be used to prove the accuracy of channel estimation by combining information from the spatial domain. By taking advantage of the fusion information, the algorithm can be optimized to better detect and locate signals in a real-world environment. Researchers have explored information fusion from multiple sources (e.g., energy domain, time domain, frequency domain, spatial domain) to enhance accuracy in complex wireless communication environments [10]. This paper proposes a novel spatial multi-path information fusion localization algorithm in ground NLOS environments. The main contributions of this paper are summarized as follows:

1. We present a spatial multi-path array fusion model. The mathematical model is constructed under the assumption of a single reflection scenario, where the positions of the localization station and reflector are known.
2. An AOA/TOA optimal joint localization algorithm for ground NLOS situations is proposed. To improve the localization accuracy, we comprehensively use the fusion information. By decomposing the signal noise subspace, the proposed algorithm fuses the multi-data from the path with array element and geospatial data.
3. Cramer–Rao Lower Bound (CRLB) was derived and used to compare the proposed algorithm with other localization algorithms, and the results shows that our algorithm achieves better estimation performance.
4. To validate the effectiveness of the proposed algorithm, we conducted simulations under different numbers of array elements and reflection paths. The results reveal that the proposed algorithm has excellent ability to measure multi-array data and has effective information fusion capability.

The rest of this paper is organized as follows: the related work is described in Section 2; in Section 3, a mathematical model for spatial multi-path array fusion localization is constructed; Section 4 implements a localization algorithm that fuses path, array element, and geospatial multi-data based on the signal-noise subspace decomposition method; Section 5 provides the CRLB for the proposed algorithm to further analyze the localization performance, with the effectiveness of the algorithm verified through simulation experiments described in Section 6; a discussion of the proposed algorithm is presented in Section 7; and finally, Section 8 concludes the paper. The notations are used in this paper are shown in Table 1.

## 2. Related Work

Researchers have investigated various localization algorithms to mitigate the negative effects of NLOS transmissions. These studies are primarily focused on suppressing or eliminating the adverse effects of non-direct paths. AOA and TOA are two common key factors in localization technologies, and many scholars have studied the related methods to eliminate the NLOS error based on those two factors. A new localization algorithm based on AOA was proposed in [11]. This algorithm uses a Bayesian probabilistic framework to estimate the target location, models the angle error as a Gaussian distribution, and performs NLOS error correction on AOA measurement. Research of TOA-based NLOS mitigation algorithms are aimed at mitigating the effects of NLOS errors on TOA measurement. One of the studies [12] advanced the TOA NLOS mitigation cooperative localization algorithm based on a topological unit, which has higher scalability and robustness, and can obtain the target node with fewer anchor nodes. Another study [9] proposed the TOA measurements localization algorithm, which uses a semidefinite programming problem to mitigate NLOS errors without the statistical information of the NLOS error. Additionally, other methods using mathematical models or filter technology have also achieved the aim of mitigating the effect of NLOS. The author in [13] presents a NLOS mitigation algorithm that employs an equality-constrained Taylor series robust least squares method to mitigate error, which does not depend on much prior environment knowledge. A linear regression model based on hybrid filtering techniques is used to estimate and correct NLOS errors in a localization algorithm proposed by another study [14]. The expression model of the error caused by NLOS and the covariance of the location error was established in [15] using small error analysis, to estimate the multi-path effect. However, while these methods effectively handle NLOS errors, they also lose the location information contained in the multi-path propagation of the signal. 

In a wireless localization system, the target location information exists not only in the direct path signal but also in the non-direct path signals. Therefore, it is necessary to explore multi-paths to obtain additional useful information regarding the location [16]. Multi-path assist positioning is a technique that uses signal propagation characteristics to estimate distance and angle. Xie indicates that multi-path signals reflect the geometry information and target location, and proposed a localization method based on the collaboration of multiple base stations [17]. Multi-path signal information is also be used in [18] to develop a searching algorithm to estimate the location of the target, and the simulation result in the actual environment shows that the median location error is about 1.5. A further study [19] proposed a multipath-assisted localization method, considering the scattering as Gaussian distribution in NLOS environments, using AOA and information from multi-paths to calculate the scatterers with different weights, and finally, optimizing the localization objective function and obtaining the final result of the estimated position. Multi-path data still has some limitations, and they may not be sufficient to support information accuracy, because the number, distribution, and strength of the signal may vary with environmental changes. Therefore, it is necessary to fuse more data to assist positioning.

Data fusion has become a popular topic in recent research in the wireless localization field as single domain information is unable to meet the increasing demand for accuracy. An information fusion method was proposed in reference [10] that utilized a maximum likelihood estimator based on energy, time, and frequency domain fusion to precisely estimate the localization of the target. The combination of spatial and time parameters is also an active research area for target localization. The author of [20] used multi-sensor combined received signal strength (RSS), AOA, and TOA for single target localization, and optimized sensor placement strategies to obtain the best estimate result. The time difference of arrival (TDOA) and AOA fusion algorithm were developed in [21] by introducing a constrained total least squares framework. The proposed algorithm had better performance in both localization accuracy and robustness. The target position was estimated in [22] using multi-source information fusion in 6G location-based services via Gaussian kernel density estimation. Multi-source RSS information was merged in line with Dempster–Shafer evidence theory, and the ideal reference points were chosen as the target position. This method has advantages in processing incomplete RSS information. A multi-source information fusion algorithm was also proposed in another study [23]. Information from sensors and geometric data of the building were fused, and an adaptive complementary filtering algorithm was used to obtain the estimated information, which contributed to a higher accuracy of orientation. A fusion of pedestrian dead-reckoning (PDR) and ultra-wideband (UWB) was utilized in [24] based on incremental smoothing. Additionally, the relative merits of the Huber kernel and Tukey kernel were compared, and the proposed method achieved high-accuracy positioning under NLOS conditions. Angular and distance information were combined for localization in [25] to evaluate single base station positioning performance in a practical environment. The TOA information in the 4G network rounding reference signal was used to estimate the distance, and the AOA information was obtained using the multi-signal classification (MUSIC) algorithm. The results showed a median location error of around 2 m. The author in [26] demonstrated that estimates of the position of an unknown node can be determined using data fusion of AOA and path loss. The bandwidth of 800 MHz showed better localization performance with the proposed algorithm. 

Other studies [27,28,29] have shown that data fusion combined with RSS can enhance overall positioning performance. Machine learning was used as a solution in [27], where the proposed neural network model obtained better localization performance by fusing the position data in GNSS and user equipment position from RSS. The results showed the localization error was reduced by up to 49%. Direction of arrival (DOA) and TOA were fused with RSS by the authors in [28], and an iterative algorithm using RSS and TOA was used in [29]. The non-convex estimation problem was converted into a generalized trust region subproblem to solve the problem in directional target localization in NLOS environments.

## 3. Spatial Multi-Path Array Fusion Localization Model

In this section, we present the spatial multi-path array fusion localization model, which is used to construct a mathematical model of the received signal, including the AOA and TOA information of the signal, to improve localization accuracy.

**Figure 1:**
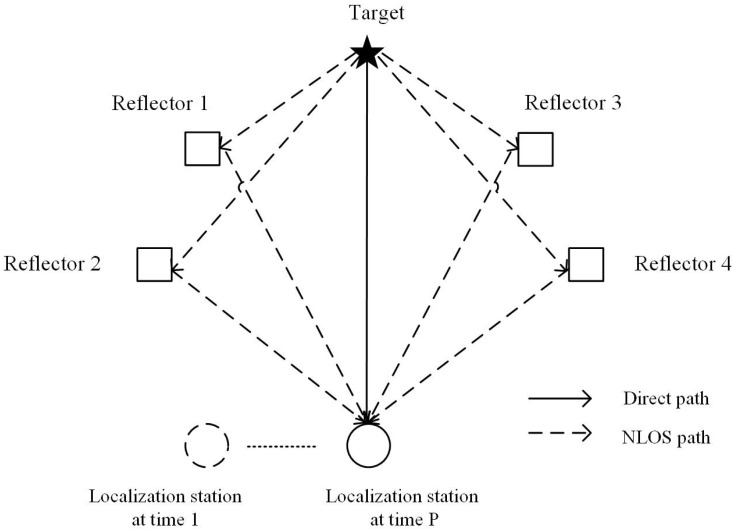
Single reflection in multi-path localization scenario.

Signal attenuation can occur in a NLOS environment where the signal is being reflected off of obstacles, such as walls, trees, and buildings. With each reflection, the signal experiences a decrease in power, resulting in a weaker signal at the receiver that may be too weak to observe. Hence, this paper focuses on the multi-path localization scenario with a single reflection, as shown in Figure 1, where the signal reflects only once for each reflector. In this scenario, if the reflector is far from both the target and the localization station, the distance is much greater than the wavelength of the signal. In this case, the reflector appears as a single point in the localization system, and the reflector’s size and shape have a negligible effect on the signal. Therefore, the reflector’s position can be used in the location calculation, instead of the reflection point of the electromagnetic wave. This approach simplifies the localization process. In practical applications, the reflector position can be obtained through measurement or by referring to a geographic information system (GIS).

The construction of a multipath signal reception model is an important step in developing accurate localization algorithms for NLOS environments. In the case of considering the reflector as a point, the multi-path signal reception model can be constructed as follows.

Assume that the localization station consists of a linear array of M elements, and the distance between every array element is $d_{m}$. The localization station receives the target signal P times, the coordinate of the localization station is $u_{p}^{}=(u_{p,x},u_{p,y}),p=1,2,\cdots ,P$. The model assumes that the target signal reaches the localization station through one direct path and L reflection paths. The reflection point coordinate of each reflection path is $\mu_{l}^{}=(\mu_{l,x},\mu_{l,y}),l=1,2,\cdots ,L_{}$, and the coordinate of the target to be estimated is $\nu_{}^{}=(\nu_{x},\nu_{y})$, therefore, the received signal $r_{p}$ of the localization station at p time can be expressed as follows:

(1) $$r_{p}(t)=\sum_{l=0}^{L}\beta_{l}a_{l}(\theta_{l}^{})s(t-\tau_{l}-t_{}^{\left(0\right)})+w(t)$$

where the parameter with subscript l indicates the relevant parameter of the l th target path, l=0 represents the direct path; $\beta_{l}^{}$ is the channel plural fading factor of the l th path; $\theta_{l}^{}$ is the angle of incidence of the l th path; $\tau_{l}$ is the time delay of the l th path; $t_{}^{\left(0\right)}$ is the launch time of the target; s is the target transmit signal; $a_{l}^{}$ indicates array manifold vector of the localization station; and w(t) is uncorrelated zero-mean Gaussian white noise with variance $\sigma_{w}^{2}$.

Suppose the total reception time of the signal is T and divide it into K parts, where each part has a length of T/K. By taking a sample at each part with a period of $T_{s}$, a total of N sampling points can be obtained. Hence, Equation (1) can be expressed as:

(2) $$\begin{array}{l} r_{p}(n,k)=\sum_{l=0}^{L}\beta_{l}^{}a_{l}^{}(\theta_{l}^{})s(nT_{s}-\tau_{l}-t_{}^{\left(0\right)},k)+w(n,k)\\ \;\;\;\;\;\;\;\;\;\;\;\;\;\;\;\;\;\;\;\;\;\;\;\;\;\;\;\;\;\;n=1,2,\cdots ,N,k=1,2,\cdots ,K \end{array}$$

Taking the Fourier transform to $r_{p}(n,k)$ for every *K* part, and adding the time delay information, gives us:

(3) $${\tilde{r}}_{p}(n,k)=\sum_{l=0}^{L}\beta_{l}^{}a_{l}^{}(\theta_{l}^{})e_{}^{-j2\pi n\tau_{l}/NT_{s}}\tilde{s}(n,k)e_{}^{-j2\pi nt_{}^{\left(0\right)}/NT_{s}}+\tilde{w}(n,k)$$

where $\tilde{s}$ and $\tilde{w}$ represent the Fourier coefficients of the signal and noise, respectively. Substituting Equation (2) into Equation (3), we obtain:

(4) $$\left\{\begin{array}{l} {\bar{a}}_{l}(n,\nu )=a_{l}(\theta_{l})e^{-j2\pi n\tau_{l}/NT_{s}}\\ A(n,\nu )=[{\bar{a}}_{0}(n,\nu ),{\bar{a}}_{1}(n,\nu ),\cdots ,{\bar{a}}_{L}(n,\nu )]\\ \beta ={\left[\beta_{0},\beta_{2},\cdots ,\beta_{L}\right]}^{T}\\ \bar{S}(n,k)=\tilde{s}(n,k)e^{-j2\pi nt_{}^{\left(0\right)}/NT_{s}} \end{array}\right.$$

Equation (3) can be represented as:

(5) $$\;\;{\tilde{r}}_{p}(n,k)=A(n,\nu )\beta \bar{S}(n,k)+\tilde{w}(n,k)=\Phi (n)\bar{S}(n,k)+\tilde{w}(n,k)$$

where:

(6) Φ(n)=A(n,ν)β

By constructing this multi-path signal reception model, the proposed algorithm in the next section can estimate the location of the target using the received signals, the known position of the localization station, and the position of the reflector. This model serves as a basis for developing the proposed spatial multi-path array fusion localization algorithm.

## 4. Spatial Multi-Path Array Fusion Localization Algorithm

In this section, a novel localization algorithm for ground NLOS situations was proposed. The algorithm is based on a joint localization method that incorporates spatial information from multiple sources, AOA, and TOA to improve the localization accuracy. 

Using conditions in which the reflector point model considers the reflector as a point, there is an unknown bias $\Delta \mu_{l}^{}$ between the measured position of the reflector and its actual position. The bias is caused by the fact that the reflector is not a point, but rather a physical object with a finite size. As a result, the measured position of the reflector is not the same as its actual position. The actual coordinates of the reflector can be expressed as ${\bar{\mu }}_{l}^{}=\mu_{l}^{}+\Delta \mu_{l}^{}$. The bias $\Delta \mu_{l}^{}$ is called the point error. The angle and time delay parameters of the signal after reflect denoted as ${\bar{\theta }}_{l}$ and ${\bar{\tau }}_{l}$, respectively, and they can be written as:

(7) $$\left\{\begin{array}{l} {\bar{\theta }}_{l}^{}=\theta_{l}^{}+\Delta \theta_{l}^{}\\ {\bar{\tau }}_{l}=\tau_{l}+\Delta \tau_{l} \end{array}\right.$$

where $\theta_{l}$ and $\tau_{l}$ are the signal angle and time delay calculated from the measured value of the reflector position; $\Delta \theta_{l}$ and $\Delta \tau_{l}$ are the angle and time error caused by the point error. By incorporating Equation (7) into (2), the signal model with the error is obtained as:

(8) $$r_{p}(n,k)=\sum_{l=1}^{L}\beta_{l}^{}a_{l}^{}({\bar{\theta }}_{l}^{})s(nT_{s}-{\bar{\tau }}_{l}-t_{}^{\left(0\right)},k)+\beta_{0}^{}a_{0}^{}(\theta_{0}^{})s(nT_{s}-\tau_{0}-t_{}^{\left(0\right)},k)+w(n,k)$$

It is assumed that the reflector is distant from the localization station and the target, and as a result the bias error of the reflector position is small, and $\Delta \tau_{l}$ can be combined into the signal’s plural decay. Applying the Fourier transform to $r_{p}$ gives us:

(9) $${\tilde{r}}_{p}(n,k)=\left(\sum_{l=1}^{L}{\overset{⌢}{\beta }}_{l}^{}a_{l}^{}({\bar{\theta }}_{l}^{})e_{}^{-j2\pi n\tau_{l}/NT_{s}}+\beta_{0}^{}a_{0}^{}(\theta_{0}^{})e_{}^{-j2\pi n\tau_{0}/NT_{s}}\right)\cdot \tilde{s}(n,k)e_{}^{-j2\pi nt_{}^{\left(0\right)}/NT_{s}}+\tilde{w}(n,k)$$

where ${\overset{⌢}{\beta }}_{l}^{}=\beta_{l}^{}e_{}^{-j2\pi n\Delta \tau_{l}/NT_{s}}$. $a_{l}^{}({\bar{\theta }}_{l}^{})$ is the function that relates to the reflector angular bias error. The first-order Taylor series expansion is used under the assumption of small bias error, which can be represented as:

(10) $$a_{l}^{}({\bar{\theta }}_{l}^{})\approx a_{l}^{}(\theta_{l}^{})+b_{l}^{}(\theta_{l}^{})\Delta \theta_{l}^{}$$

where $b_{l}^{}(\theta_{l}^{})={\left.\frac{\partial a_{l}^{}(\theta )}{\partial \theta }\right|}_{\theta =\theta_{l}^{}}$ is the first order value of $a_{l}^{}(\theta )$ when $\theta =\theta_{l}^{}$. Combining Equation (10) with Equation (9), ${\tilde{r}}_{p}(n,k)$ can be expressed as:

(11) $$\begin{array}{ll} \;{\tilde{r}}_{p}(n,k)\approx & (\sum_{l=1}^{L}{\overset{⌢}{\beta }}_{l}^{}a_{l}^{}(\theta_{l})e^{-j2\pi n\tau_{l}/NT_{s}}+{\overset{⌢}{\beta }}_{l}\Delta \theta_{l}b_{l}(\theta_{l})e^{-j2\pi n\tau_{l}/NT_{s}}+\\ & \left.\beta_{0}^{}a_{0}^{}(\theta_{0}^{})e_{}^{-j2\pi n\tau_{0}/NT_{s}}\right)\tilde{s}(n,k)e_{}^{-j2\pi nt_{}^{\left(0\right)}/NT_{s}}+\tilde{w}(n,k) \end{array}$$

Since $\Delta \theta_{l}^{}$ is irrelevant to the target location, the relationship between the time delay, angle parameter, and the target location are:

(12) $$\begin{array}{l} \left\{\begin{array}{l} \theta_{0}^{}=\arccos\frac{\nu_{x}-u_{p,x}}{\left\|\nu -u_{p}\right\|}\\ \tau_{0}=\frac{1}{c}\left\|\nu -u_{p}\right\| \end{array}\right.\;\;\;\;\;\\ \left\{\begin{array}{l} \theta_{l}^{}=\arccos\frac{\mu_{l,x}-u_{p,x}}{\left\|\mu_{l}-u_{p}\right\|}\\ \tau_{l}=\frac{1}{c}(\left\|\nu -\mu_{l}\right\|+\left\|\mu_{l}-u_{p}\right\|) \end{array}\right.l=1,2,\ldots ,L_{} \end{array}$$

Combining Equation (12) with Equation (11), gives us:

(13) $$\left\{\begin{array}{l} {\bar{b}}_{l}^{}(n,\nu )=b_{l}^{}(\theta_{l}^{})e_{}^{-j2\pi n\tau_{l}/NT_{s}}\\ B(n,\nu )=[A(n,\nu ),{\bar{b}}_{1}^{}(n,\nu ),{\bar{b}}_{2}^{}(n,\nu ),\cdots ,{\bar{b}}_{L}^{}(n,\nu )]\\ \overset{⌣}{\beta }={\left[\beta_{0},{\overset{⌢}{\beta }}_{1},\cdots ,{\overset{⌢}{\beta }}_{L},\Delta \theta_{l}{\overset{⌢}{\beta }}_{1},\cdots ,\Delta \theta_{L}{\overset{⌢}{\beta }}_{L}\right]}^{T} \end{array}\right.$$

Thus Equation (11) can be expressed as:

(14) $$\;\;{\tilde{r}}_{p}(n,k)=B(n,\nu )\overset{⌣}{\beta }\bar{s}(n,k)+\tilde{w}(n,k)=F(n)\bar{S}(n,k)+\tilde{w}(n,k)$$

For the subspace-based fusion localization algorithm when the signal is unknown, the covariance matrix of the received signal is:

(15) $$R_{p}=E[\;{\tilde{r}}_{p}{\tilde{r}}_{p}^{H}]=F(n)R_{s}F{\left(n\right)}_{}^{H}+\sigma_{w}^{2}I_{}^{}$$

In this equation, $I_{}^{}$ is a unit matrix and $R_{s}$ is the self-covariance matrix of the signal. The subspace decomposition is performed on $R_{p}$ to obtain the noise subspace $U_{p,w}^{}$. Based on the orthogonality of the signal subspace and the noise subspace:

(16) $${\overset{⌣}{\beta }}_{}^{H}B_{}^{H}(n,\nu_{})U_{p,w}^{}U_{p,w}^{H}B_{}^{}(n,\nu_{})\overset{⌣}{\beta }=0$$

The target function can be written as:

(17) $$g_{p}^{}(\nu )=\sum_{n=1}^{N}{\overset{⌣}{\beta }}_{}^{H}B_{}^{H}(n,\nu_{})U_{p,w}^{}U_{p,w}^{H}B_{}^{}(n,\nu_{})\overset{⌣}{\beta }={\overset{⌣}{\beta }}_{}^{H}C_{p}(\nu )\overset{⌣}{\beta }$$

where $C_{p}(\nu )=\sum_{n=1}^{N}B_{}^{H}(n,\nu_{})U_{p,w}^{}U_{p,w}^{H}B_{}^{}(n,\nu_{})$. The estimated target location corresponds to the minimum value obtained from $g_{p}^{}(\nu )$. This can be expressed using the Rayleigh quotient:

(18) $$\min g_{p}^{}(\nu )=\min{\overset{⌣}{\beta }}_{}^{H}C_{p}\overset{⌣}{\beta }=\lambda_{\min}^{}(C_{p}(\nu ))$$

where $\lambda_{\min}^{}$ is the minimum value of the matrix eigenvalues. By fusing the information of the location station from P moment, the final estimate position of the target can be expressed as:

(19) $$\hat{v}=\underset{\nu }{\arg\min}\sum_{p=1}^{P}\lambda_{\min}^{}(C_{p}(\nu ))$$

where ν is the target location and $\hat{v}$ is its estimated value. Equation (19) can be used to estimate the target location. There are two advantages to this equation. The first advantage of this equation is that it does not need to consider the effect of multi-path fading, which reduces the number of estimation parameters, simplifying the algorithm and making it more efficient. Secondly, the equation use the Taylor series expansion to mitigate the impact of reflector position bias errors. This aligns with the real-world localization environment that has small errors in reflector location. 

A simulation experiment will analyze the performance of the algorithm and compare it with existing algorithms to prove its effectiveness of the algorithm.

The implementation of the proposed algorithm is described in Table 2.

## 5. Derivation of the Cramer–Rao Lower Bound

In this section, we derivate the CRLB to further analyze the localization performance of the algorithm proposed in this paper. The CRLB is a theoretical lower bound on the variance of any unbiased estimator of a parameter, and it represents the best possible performance that can be achieved by any estimator. 

Under the model that we proposed in the Section 4, the parameter to be estimated can be set as:

(20) ρ=[ν¯T,Re{β¯T},Im{β¯T},σ¯sT]T

where ${\bar{\sigma }}_{s}^{}$ represents the power of the signal. The Fisher information matrix (FIM) of the unknown parameters can be expressed as:

(21) $$J_{i,j}=\frac{K}{2}\sum_{p=1}^{P}\sum_{n=1}^{N}\mathrm{tr}\left\{R_{p}^{-1}\frac{\partial R_{p}^{}}{\partial \rho_{i}}R_{p}^{-1}\frac{\partial R_{p}^{}}{\partial \rho_{j}}\right\}\;\;\;\;\;\;\;\;\;\;$$

where $\rho_{i}$ and $\rho_{j}$ are the i th and j th parameter of the unknown vector. The covariance matrix of the received signal is expressed as:

(22) $$R_{p}^{}=F(n)R_{s}F{\left(n\right)}_{}^{H}+\sigma_{w}^{2}I_{}^{}$$

Then:

(23) $$\frac{\partial R_{p}^{}}{\partial \nu }=\frac{\partial F(n)}{\partial \nu }R_{s}^{}F{\left(n\right)}_{}^{H}+F(n)R_{s}^{}\frac{\partial F(n)}{\partial \nu }$$

Allowing ${\left[B(n,\nu )\right]}_{m,l}^{},m=1,2,\cdots ,M,l=0,1,\cdots ,2L$ to represent the number (m,l) element of the matrix, and assuming the distance between every array element $d_{m}$ equals a half wavelength, then:

(24) $$\left\{\begin{array}{l} {\left[B(n,\nu )\right]}_{m,l}=\exp(-j\pi (m-1)\cos(\theta_{l}^{}))\cdot \exp(-j2\pi f_{s}^{}\tau_{l}^{}n/N)\;\;\;\;\;\;\;\;\;\;\;l\leq L\\ {\left[B(n,\nu )\right]}_{m,l}=j\pi (m-1)\sin(\theta_{l-L}^{})\exp(-j\pi (m-1)\cdot \cos(\theta_{l-L}^{}))\exp(-j2\pi f_{s}^{}\tau_{l-L}^{}n/N)\;\;\;l>L \end{array}\right.$$

where:

(25) $$\left\{\begin{array}{l} \frac{\partial {\left[B(n,\nu )\right]}_{m,l}^{}}{\partial \nu_{x}^{}}=\left[-j\pi (m-1)\frac{\partial \cos(\theta_{l}^{})}{\partial \nu_{x}^{}}-j2\pi f_{s}^{}\frac{\partial \tau_{l}^{}}{\partial \nu_{x}^{}}n/N\right]\cdot \\ {\left[B(n,\nu )\right]}_{m,l}\;\;\;\;\;\;\;\;\;\;\;\;\;\;\;\;\;\;\;\;\;\;\;\;\;\;\;\;\;\;\;\;\;\;\;\;l\leq L\\ \frac{\partial {\left[B(n,\nu )\right]}_{m,l}}{\partial \nu_{x}^{}}=\left[\frac{1}{\sin(\theta_{l-L}^{})}\frac{\partial \sin(\theta_{l-L}^{})}{\partial \nu_{x}^{}}-j\pi (m-1)\frac{\partial \cos(\theta_{l-L}^{})}{\partial \nu_{x}^{}}-\right.\\ \left.\;j2\pi f_{s}^{}\frac{\partial \tau_{l-L}^{}}{\partial \nu_{x}^{}}n/N\right]{\left[B(n,\nu )\right]}_{m,l}\;\;\;\;\;\;l>L \end{array}\right.$$

$\frac{\partial {\left[B(n,\nu )\right]}_{m,l}^{}}{\partial \nu_{y}^{}}$ can be obtained in the same way. The expressions of the angle and time delay are different for the direct and non-direct paths. When l=0 represents the direct path, there is:

(26) $$\begin{array}{l} \left\{\begin{array}{l} \frac{\partial \cos(\theta_{l}^{})}{\partial \nu_{x}^{}}=\frac{{\left(\nu_{y}^{}-u_{p,y}^{}\right)}_{}^{2}}{d_{p}^{3}}\\ \frac{\partial \cos(\theta_{l}^{})}{\partial \nu_{y}^{}}=-\frac{\left(\nu_{y}^{}-u_{p,y}^{})(\nu_{x}^{}-u_{p,x}^{}\right)}{d_{p}^{3}} \end{array}\right.\;\;\;\;\;l=0\\ \;\left\{\begin{array}{l} \frac{\partial \cos(\theta_{l}^{})}{\partial \nu_{x}^{}}=0\\ \frac{\partial \cos(\theta_{l}^{})}{\partial \nu_{y}^{}}=-0 \end{array}\right.\;\;\;\;\;\;\;\;\;\;\;\;\;\;\;\;\;\;\;\;\;\;\;\;\;\;\;l\neq 0 \end{array}$$

(27) $$\left\{\begin{array}{l} \frac{\partial \tau_{l}^{}}{\partial \nu_{x}^{}}=\frac{\left(\nu_{x}^{}-u_{p,x}^{}\right)}{cd_{p}^{}}\\ \frac{\partial \tau_{l}^{}}{\partial \nu_{y}^{}}=\frac{\left(\nu_{y}^{}-u_{p,y}^{}\right)}{cd_{p}^{}} \end{array}\right.\;\;\;\;\;\;\;\;\;\;\;l=0;\;\;\;\;\;\;\;\;\;\;\;\left\{\begin{array}{l} \frac{\partial \tau_{l}^{}}{\partial \nu_{x}^{}}=\frac{\left(\nu_{x}^{}-\mu_{l,x}^{}\right)}{cd_{l}^{}}\\ \frac{\partial \tau_{l}^{}}{\partial \nu_{y}^{}}=\frac{\left(\nu_{y}^{}-\mu_{l,y}^{}\right)}{cd_{l}^{}} \end{array}\right.\;\;\;\;\;\;l\neq 0$$

The derivative for the multi-path attenuation of the target signal is:

(28) ∂Rp∂Re(β⌣T)=∂F(n)∂Re(β⌣T)RsF(n)H+F(n)Rs∂F(n)∂Re(β⌣T)

where:

(29) ∂F(n)∂Re(β⌣T)=[0,0,⋯,B(n,ν)∂β⌣∂Re(β⌣T),⋯,0]

Then there is:

(30) ∂β⌣∂Re(β⌣T)=[0,0,⋯,0,1,0,⋯,0]

Only the l th element is 1 and the remaining are 0. Therefore:

(31) ∂Rp∂Im{β⌣T}=j∂Rp∂Re(β⌣T)

where:

(32) $$\frac{\partial R_{p}^{}}{\partial \sigma_{s}^{2}}=F(n)\frac{\partial R_{s}^{}}{\partial \sigma_{s}^{2}}F{\left(n\right)}_{}^{H}$$

(33) $$\frac{\partial R_{s}^{}}{\partial \sigma_{s}^{2}}=[0,0,\cdots ,1,\cdots ,0,0]$$

Given the above derivation, we can obtain the derivative $\frac{\partial R_{p}^{}}{\partial \rho_{i}}$ for any element of ρ. FIM can be obtained according to Equation (21), and thus we can express CRLB as:


(34) $$CBLB=J^{-1}$$

The CRLB can be derived by calculating the inverse of the FIM, which provides a lower bound on the variance of any unbiased estimator of the target location. The CRLB can be used to compare the performance of different localization algorithms and to evaluate the efficiency of our proposed algorithm. 

## 6. Algorithm Simulation and Verification

In this section, the performance of our proposed spatial multi-source information fusion localization algorithm (IFLA) is evaluated and compared to the traditional two-step localization algorithm. The traditional AOA/TOA localization algorithm is based on the least square method (ML_AOA/TOA), and estimates the location of a target by combining AOA and TOA information, and utilizes the multiple signal classification (MUSIC) algorithm for AOA parameter estimation and the maximum likelihood time-delay estimation algorithm for TOA parameter estimation [30]. After obtaining the AOA and TOA parameters, the target localization is achieved through the least squares method [30]. 

In the simulation, two targets are placed in the plan-coordinate system with coordinates of (500, 3500) m and (−2000, 2800) m. Three localization stations are placed at coordinates (−3000, −1000) m, (0, −1000) m, and (3000, −1000) m, to receive signals from the target. Additionally, two reflectors are located in the localization area with coordinates of (−4000, 1000) m, (4000, 1000) m. The simulation parameters include an initial number of array elements (7), the received signal divided into 32 segments with each segment having 16 sampling points, a signal carrier frequency of 1 GHz, and the sampling frequency of 0.5 MHz. The parameters described above are shown in Table 3.

**Figure 2:**
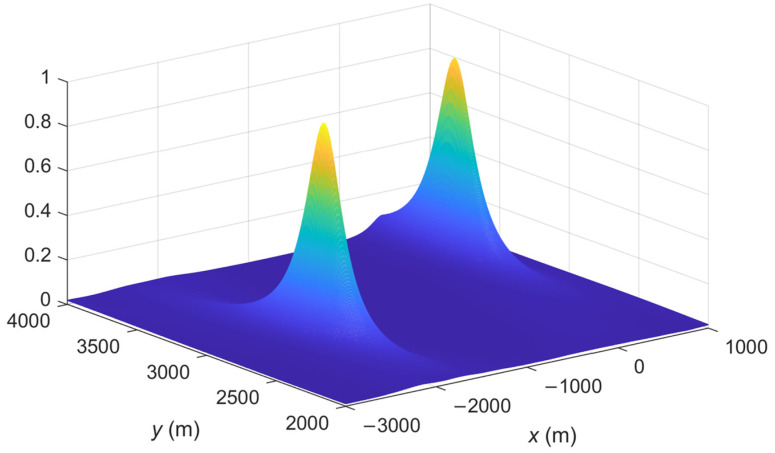
Spatial spectrum of ML_AOA/TOA (stereogram).

**Figure 3:**
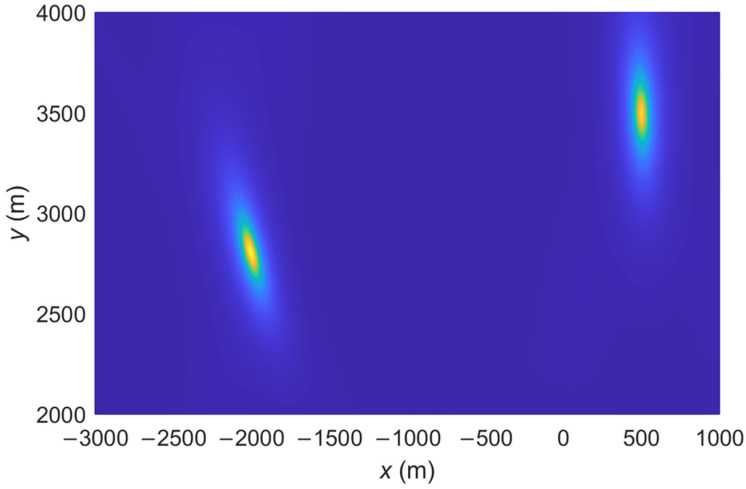
Spatial spectrum of ML_AOA/TOA (top view).

**Figure 4:**
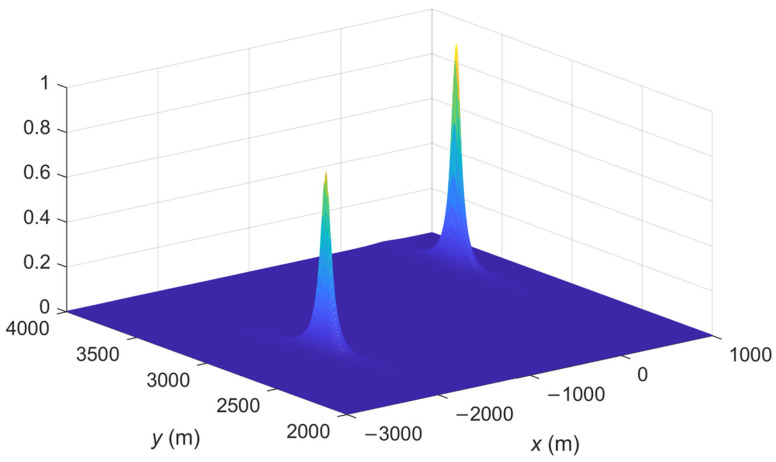
Spatial spectrum of IFLA (stereogram).

**Figure 5:**
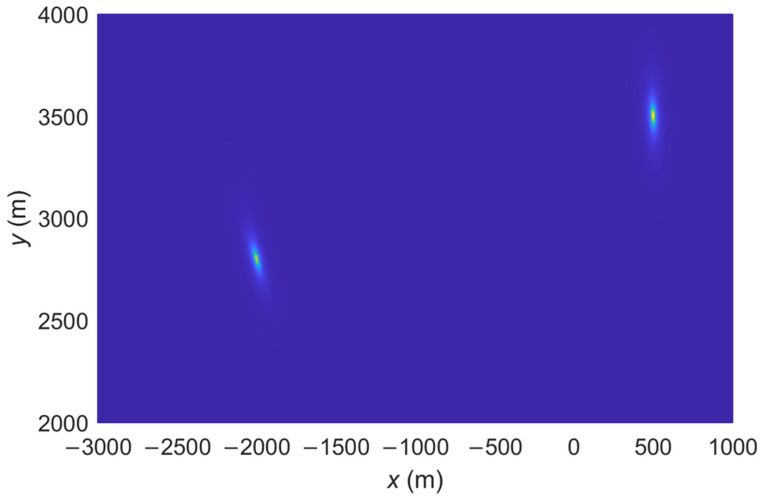
Spatial spectrum of IFLA (top view).

The spatial spectrum of the proposed IFLA algorithm and ML_AOA/TOA algorithm are compared. The spatial spectrum is a common tool used in signal processing to analyze and visualize the spatial spectrum to analyze the accuracy of a localization algorithm. A sharp peak in the spatial spectrum plot indicates that the signal has a strong spatial frequency component at that particular frequency. This can be interpreted as the presence of a spatial pattern or feature in the signal at that frequency. To demonstrate the effectiveness of the proposed IFLA algorithm, Figure 2, Figure 3, Figure 4 and Figure 5 shows the stereogram and top view of the spatial spectrum of both the IFLA and ML_AOA/TOA algorithms with a signal to noise ratio (SNR) of 0 dB. The simulation results indicate that the IFLA achieves a sharp peak at the true position of the target with a smaller fuzzy area, which indicates that IFLA has a higher localization accuracy compared to ML_AOA/TOA.

To further illustrate the performance of IFLA algorithms, we contrast the localization performance of each algorithm with their CRLBs. The CRLB and root mean square error (RMSE) of each algorithm were obtained through 500 Monte Carlo simulation experiments as a function of SNR. The SNR was calculated using the received signal power and the noise power, as shown in Equation (35). The formula for RMSE is show in Equation (36), where N is the number of Monte Carlo simulations, ${\hat{y}}_{i}$ represent the estimate value of the target and $y_{i}$ represent the real value of it:

(35) $$SNR=10\mathrm{lg}(\frac{P_{signal}}{P_{noise}})(dB)$$

(36) $$RMSE=\sqrt{\frac{1}{N}\sum_{i=1}^{n}{\left({\hat{y}}_{i}-y_{i}\right)}^{2}}$$

**Figure 6:**
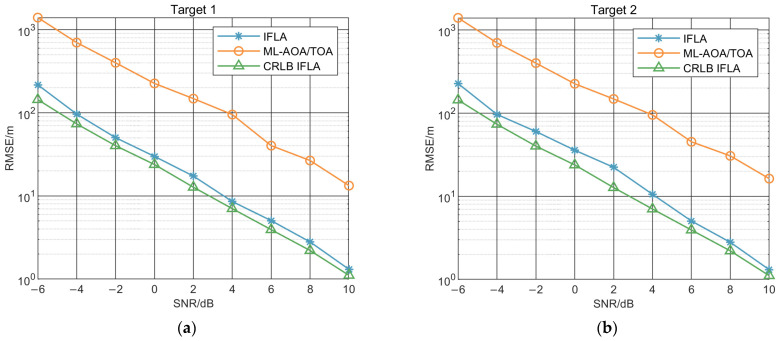
Estimation accuracy comparison of IFLA (blue) and ML_AOA (orange)/TOA (green) for target 1 (**a**) and target 2 (**b**).

The results are presented in Figure 6 and show that both IFLA and the ML_AOA/TOA improve with increasing SNR. However, the IFLA outperforms the ML_AOA/TOA throughout the entire range of SNR values. These results indicate that the IFLA algorithm is able to provide more accurate localization of the target.

**Figure 7:**
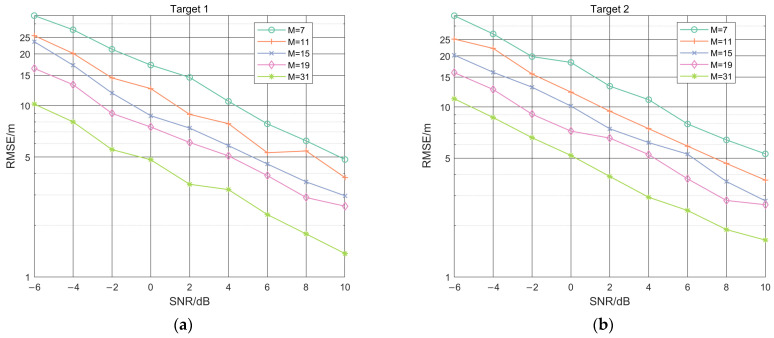
IFLA Localization performance comparison with different numbers of array elements (M), for target 1 (**a**) and target 2 (**b**).

To verify the fusion localization performance of the IFLA for antenna array element data, and to further simulate the error under a different number of array element, IFLA was used to perform 500 Monte Carlo simulation experiments for different numbers of array elements (M), including 7, 11, 15, 19, and 31. The results in Figure 7 show that when the value of SNR is constant, the RMSE decreases with the increase of the array element number, which indicates that the localization performance of IFLA improves as the number of array elements increases. This simulation also demonstrates the IFLA algorithm has effective information fusion capability for measuring multi-array element antenna data.

**Figure 8:**
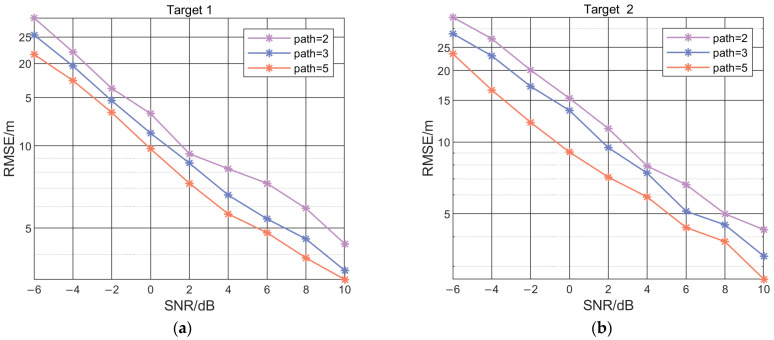
IFLA Localization performance comparison with different numbers of signal propagation paths (path) for target 1 (**a**) and target 2 (**b**).

To verify the fusion localization performance of the IFLA for the number of spatial propagation paths, and to further simulate the error with a different number of signal paths, IFLA was used to perform 500 Monte Carlo simulation experiments with a different numbers of reflectors, represent as paths, including 2, 3, and 5 reflectors. The results in Figure 8 shows that when the value of SNR is the same, the RMSE decreases with the increase of reflection paths, which indicates that the localization performance of IFLA improves as the number of signal reflection paths increases. This simulation results also demonstrate that the algorithm has effective information fusion capability for spatial signal multi-path data.

## 7. Discussion

In NLOS environments, traditional localization algorithms face challenges, such as multipath fading, signal attenuation, and interference, which degrade the accuracy of the localization process. Spatial multi-path information fusion has been proposed as a promising approach to address the challenges of NLOS localization. This approach fuses multi-array element antenna data and spatial signal multi-path data to improve localization accuracy. The IFLA algorithm proposed in this paper utilizes this approach and outperforms the traditional ML_AOA/TOA algorithm, and has effective information fusion capability for measuring multi-array element antenna data and spatial signal multi-path data. However, despite its advantages, the accuracy of the localization process is still restricted by various external factors. The complexity of the environment and the accuracy of prior environmental information, as well as the signal bandwidth, SNR, and data sampling time, can all affect the algorithm’s performance. Moreover, the IFLA algorithm is limited to recognizing only one target in a 2D plane and relies on assumptions about the environment, such as the existence of only one reflection path for one reflector. In the real-world scenarios, the presence of multiple targets may interfere with the target to be located, and the IFLA algorithm may not be applicable. 

## 8. Conclusions

This paper presents a novel algorithm aimed at improving localization accuracy in ground NLOS environments. A spatial multi-path information fusion localization model is proposed by analyzing NLOS error mitigation localization algorithms. The model is further improved by incorporating path, array element, and geospatial multi-data using the signal noise subspace decomposition in a fusion localization algorithm. The CRLB of the algorithm is derived, which shows the localization accuracy is better compared with the existing traditional algorithm. Simulation experiments are conducted to verify the effectiveness of the proposed localization algorithm. The results show that the proposed method effectively leverages spatial multi-source information to achieve precise localization in complex ground environments and improves the locatable probability. This algorithm can be applied in various localization scenarios, such as indoor, complex urban environments, factories, underground tunnels, and other applications scenarios. Future research directions include investigating the 3D localization, multi-target scenarios, dynamic target scenarios, and studying the mutual influence between multiple targets and the localization station on the localization performance. 

## Figures and Tables

**Table 1:** Notations.

Transpose of matrix	${\left[•\right]}^{T}$
Hermitian transpose of matrix	${\left[•\right]}^{H}$
Identity matrix	I
(*m,n*)-th entry of a matrix	${\left[•\right]}_{m,n}$
The real part of the complex number *x*	Re{x}
The imaginary part of the complex number *x*	Im{x}

**Table 2:** The proposed algorithm steps.

**Step 1**	Calculate the covariance matrix of the received signal using Formula (15).
**Step 2**	Perform subspace decomposition on the covariance matrix $R_{p}$ to obtain the noise subspace $U_{p,w}$.
**Step 3**	Construct the target function by utilizing the orthogonality of the signal subspace and the noise subspace using Formula (17).
**Step 4**	Obtain the estimate value of the target position by finding the minimum value of the target function using Formula (18).
**Step 5**	Combine the information form *P* moments of the localization station to obtain the final estimate value using Formula (19).

**Table 3:** Simulation parameters.

Parameter	Unit	Value
Target coordinates	m	(500, 3500) and (−2000, 2800)
Localization station coordinates	m	(−3000, −1000), (0, −1000), and (3000, −1000)
Reflector coordinates	m	(−4000, 1000) and (4000, 1000)
Initial number of array elements	-	7
Number of segments for received signal	-	32
Number of sapling points per segment	-	16
Signal carrier frequency	GHz	1
Sampling frequency	MHz	0.5

## Data Availability

The data that support the findings of this study are available upon request from the authors.
